# Supercontinuum generation by co-filamentation of two color femtosecond laser pulses

**DOI:** 10.1038/s41598-019-45357-y

**Published:** 2019-06-21

**Authors:** M. Vengris, N. Garejev, G. Tamošauskas, A. Čepėnas, L. Rimkus, A. Varanavičius, V. Jukna, A. Dubietis

**Affiliations:** Laser Research Center, Vilnius University, Sauletekio Avenue 10, LT-10223 Vilnius, Lithuania

**Keywords:** Optics and photonics, Physics

## Abstract

In this paper, we experimentally investigate supercontinuum generation via collinear two-color filamentation in sapphire crystal, by launching two femtosecond pulses at fundamental (1030 nm) and second harmonic (515 nm) wavelengths from an amplified Yb:KGW laser. By changing the time delay between the incident pulses, we observe dramatic changes in the supercontinuum spectrum, transmitted energy, position of the nonlinear focus and intensity distribution along the filamentinduced luminescence traces. In particular, we show that at some delays the two pump wavelengths can assist each other in generating supercontinuum, whilst at other delays large portions of the supercontinuum spectrum are completely extinguished. The transition between supercontinuum generation and its extinction occurs within a very short (20 fs) span of the delay times, despite the fact that the pump pulses are 220 fs long. We propose that the observed non-trivial spectral dynamics can be interpreted by a mechanism, where co-propagating two pump pulses perturb the nonlinear refractive properties of the medium via Kerr effect and generation of free electron plasma thereby affecting pulse splitting and pulse front steepening, which are the key players in the process of supercontinuum generation in a normally dispersive medium.

## Introduction

Filamentation of intense femtosecond laser pulses in transparent dielectric media is a universal nonlinear propagation regime, which gives rise to large scale spectral broadening, termed supercontinuum (SC) generation^1^. SC generation in solid-state media constitutes a simple, compact and efficient method for production of ultrabroadband radiation at various parts of the optical spectrum, see^2^ for an explicit review. Owing to its high spatial and temporal coherence, SC is widely used as a seed source in optical parametric amplifiers^3^, and as a probe or even pump light in a variety of time-resolved spectroscopic experiments, see e.g.^4–6^. For such applications, it is important that SC covers as broad spectral range as possible, which becomes especially problematic in the UV, as due to fundamental limitations imposed by the ratio between the bandgap and the energy of incident photon^7^, and the chromatic dispersion of nonlinear medium^8^.

Interactions between two filament-forming beams open new perspectives for in-flight control of filament propagation, as demonstrated employing various interaction geometries in gaseous^9–12^ and solid-state^13–15^ media. Even more possibilities are offered by the so-called two-color filamentation, i.e. the nonlinear propagation regime when two ultrashort laser pulses with different carrier wavelengths are launched into the nonlinear medium at the same time. This specific filamentation regime was widely studied in gases, such as air, argon, neon and nitrogen. Co-filamentation of femtosecond laser pulses in gases was proposed as a powerful tool for controlling the collapse distance and the supercontinuum spectrum^16^. A considerable enhancement of SC spectral intensity was also reported using a weak seed at the wavelength different from the pump^17^. Two-color filamentation was demonstrated to produce intense broadband emissions at various parts of the electromagnetic spectrum, e.g. excitation of high harmonics supercontinuum in the extreme ultraviolet^18^, enabling multi-cycle laser field synthesis for delivery of isolated intense attosecond pulses^19^, production of ultrabroadband supercontinuum, which extends into the mid- and far-infrared^20^, and generation of high-energy, high average power and broadband radiation in the terahertz (sub-millimetric) frequency range^21,22^. Two-color filamentation provides bridging of the SC spectra generated by the individual filaments, and in the time domain counteracts pulse splitting, resulting in simultaneous compression of the interacting pulses down to few optical cycle duration at both carrier wavelengths^23,24^. Four-wave mixing between the filamenting pulses allows generation of broadband, fully compressible pulses in the ultraviolet^25^, wavelength-tunable pulses in the visible^26^, single cycle^27^ or even half-cycle pulses in the mid-infrared^28^, as well as production of tunable broadband pulses in the far infrared^29^.

So far, only a few studies were devoted to the investigation of two-color filamentation in condensed media. The early studies with picosecond laser pulses demonstrated the spectral broadening of a weak pulse via cross- and induced-phase modulation in the presence of an intense pulse^30^. In the femtosecond regime, energy transfer from the pump filament and co-filamentation of a weak seed was studied in the presence of gain mechanisms, such as four wave mixing and stimulated Raman scattering^31,32^. Dual wavelength pumping in a collinear interaction geometry yielded enhancement of the SC intensity, also resulting in a flatter spectrum of ultrabrodband SC^33^. Enhancement or suppression of the SC generation in a non-collinear geometry by changing the relative delay between the two interacting pulses was demonstrated as well^34^. More recently, the control of polarization of the SC spectrum was demonstrated by changing the angle between the polarization directions of two femtosecond pump pulses in an optically isotropic nonlinear medium^35^.

In this paper we experimentally investigate SC generation via collinear two-color filamentation in sapphire crystal, by launching two femtosecond pulses at fundamental (1030 nm) and second harmonic (515 nm) wavelengths from an amplified Yb:KGW laser system. By changing the time delay between the incident pulses, we observe dramatic changes in the SC spectrum, transmitted energy, position of the nonlinear focus and intensity distribution along the filament-induced luminescence traces. We also demonstrate that the SC generation and control of its spectral coverage to a certain degree is possible even in the case when the energies of both interacting pulses are set below the filamentation and SC generation threshold.

## Methods

**Figure 1:**
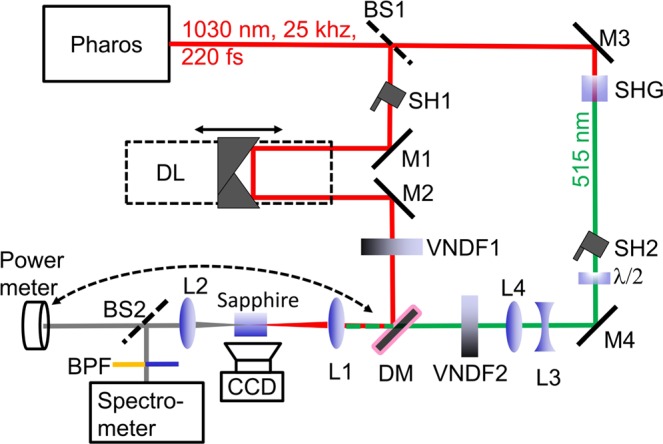
Experimental setup. BS1, BS2: beamsplitters; SH1, SH2: shutters; M1-M4: mirrors; L1-L4, lenses; SHG: second harmonic crystal; *λ*/2: half-wave plate; VNDF1, VNDF2: variable neutral density filters; DL: delay line; BPF: band-pass filters.

The experiments were performed using a setup designed to allow simultaneous measurements of the SC spectra and energy transmitted through the nonlinear crystal and at the same time monitoring the side image of the filament-induced luminescence trace. The optical layout of the setup is depicted in Fig. 1. An amplified Yb:KGW laser (Pharos, Light Conversion) operating at 25 kHz repetition rate and providing 220 fs pulses with a central wavelength of 1030 nm was used as the driving source. The laser output was split into two parts by a beamsplitter BS1. The reflected part was delayed in a delay line DL consisting of a motorized translation stage (Standa 8MT167-100) and a hollow corner cube retroreflector. The transmitted part was frequency doubled in a 1.3 mm-thick *β*-BBO crystal. The polarization of the second harmonic was rotated with a half-wave plate, *λ*/2, to make it parallel to the polarization of fundamental beam.

The co-polarized fundamental (1030 nm) and second harmonic (515 nm) beams were superimposed onto each other in space and made to co-propagate by means of a dichroic mirror DM. Lens L1 (*f* = +100 mm) focused both beams onto a 4 mm-thick sapphire crystal, the optical axis of which was aligned to be collinear with the beam propagation direction. Separate neutral density filters VNDF1 and VNDF2 allowed independent energy (power) control of the incident beams, while a telescope consisting of lenses L3 and L4 inserted into the path of the second harmonic beam allowed to ensure almost perfect matching of the focal spots of both incident beams (~30 *μ*m FWHM diameter). The SC radiation was collected by a lens L2 (*f* = +50 mm) and split into two parts using a 1° fused silica wedge beamsplitter BS2. A reflection from its first surface was directed to a spectrometer (Avantes AvaSpec-3648), the transmitted portion was coupled into a power meter (Nova 2, thermopile sensor 3 A, Ophir Optronics). To increase the dynamic range and prevent the saturation of the spectrometer, the SC spectra were collected using an integration time of 10 ms (averaging over 250 laser shots) and employing two bandpass filters attenuating most of the pump radiation at fundamental and second harmonic wavelengths. The spectral data was subsequently corrected for different filter transmissions, integration times and sensitivity function of the spectrometer throughout the overall detection range of 300–1100 nm.

The filament-induced luminescence was observed from the polished sapphire sample side parallel to the the beam propagation axis and luminescence traces were captured by imaging with 1:1 magnification onto the CCD camera (Point Grey Chameleon-2) with a pixel size of 3.75 *μ*m. Zero delay between the incident pulses was determined by the peak of the third harmonic signal generated in a thin (0.5 mm) *β*-BBO crystal, which was placed in the location of the sapphire sample. In all the subsequent presentations of the data, negative delays correspond to the fundamental (1030 nm) pulse arriving later than the second harmonic (515 nm) pulse.

## Results

### Independently generated supercontinuum spectra

**Figure 2:**
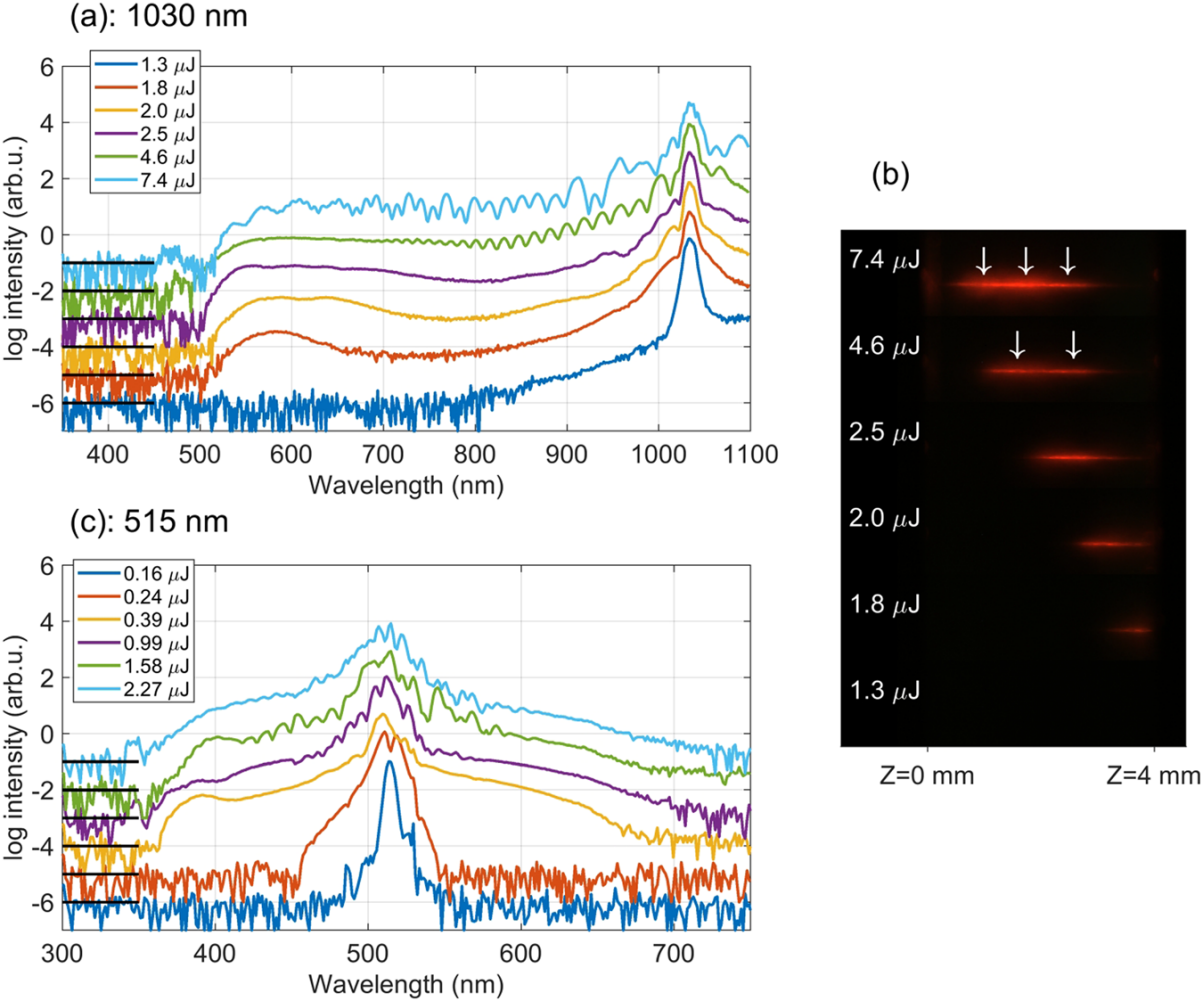
Energy dependence of SC spectra generated by (**a**) fundamental, (**c**) second harmonic pulse. For clarity, the spectra are offset by the amount indicated by black lines. The SC spectra corresponding to specific input energies are color coded and energy values are provided in legends. (**b**) Images of the luminescence traces induced by filamentation of the fundamental pulse with energies corresponding to spectral measurements. Arrows indicate multiple nonlinear foci.

To start with, we performed a brief study of the SC generation by separately launched fundamental and second harmonic pulses. First of all, we experimentally measured the threshold energies for filamentation and SC generation for fundamental and second harmonic pumps, which were 1.72 *μ*J and 0.24 *μ*J, respectively. Figure 2(a) presents the SC spectra as functions of the pump energy produced by the fundamental harmonic pulse (1030 nm) alone. Only the blue-shifted portion of the SC was recorded due to limited detection range of the spectrometer. However, since the external focusing was relatively tight (NA = 0.013), the red-shifted spectral broadening was rather modest (see ref.^36^ for details), with the long-wavelength cut-off around 1200 nm, as verified by complementary measurements using a home-built scanning prism spectrometer with Si and Ge detectors (see Supplementary Fig. S1). Therefore just a small portion of the most red-shifted SC spectrum has been left out of the detection range. Smooth and featureless SC spectrum with the short-wave cut-off at 500 nm (at the 10^−4^ intensity level) was recorded with the input energy slightly above the filamentation threshold (1.8 *μ*J). The image of the filament-induced luminescence trace shown in Fig. 2(b) attests that the input beam self-focuses into a single filament, whose the most intense part marks the position of the nonlinear focus near the exit face of the crystal. A further increase of the input energy produces the nonlinear focus closer to the input face of the sample, while the general shape of the SC spectrum and its width remain fairly constant, see the SC spectra recorded with 2.0 and 2.5 *μ*J. With the input energy of 4.6 *μ*J, refocusing of the filament was observed, producing two nonlinear foci inside the sample [indicated by arrows in Fig. 2(b)]. The two SC sources interfere producing a periodic modulation in the SC spectrum. Eventually, with the input energy of 7.4 *μ*J, filament undergoes yet another refocusing cycle that produces beatings in the modulated SC spectrum, very much in line previously described connections between the recurrent events of focusing, pulse splitting and SC generation^37^.

With the second harmonic (515 nm) pulse alone, the measured SC spectrum covers the wavelength range from 360 to 700 nm, in line with the results of an earlier experiment performed under similar conditions^38^. The spectral evolution versus the input energy is illustrated in Fig. 2(c), showing the occurrence of spectral modulation due to filament refocusing for the input energies above 1 *μ*J. In the present case, beam filamentation and SC generation were produced with a considerably lower input power, as due to the *λ*^2^ dependence of the critical power for self-focusing and dispersion of the nonlinear refractive index of sapphire^39^.

### Supercontinuum spectra produced by two-color filamentation

For the investigation of SC generation by co-filamentation of both input beams, the input energies of 0.4 *μ*J at 515 nm and 2.0 *μ*J at 1030 nm were chosen so as to produce stable SC spectra and the individual nonlinear foci close to the exit face of the sample, if the input beams/pulses are launched separately, see Fig. 2.

When the two pulses are well separated in time (few ps and more), the resulting SC spectrum is virtually identical to the sum of the SC spectra produced by the individual pulses. Interestingly, the shape of the SC spectrum at large delays shows a slight dependence on the time ordering of the pulses, indicating that the “memory” of the pulse that has passed the nonlinear medium is retained for at least a couple of picoseconds. This could be attributed to a relatively long lifetime of free electron plasma in sapphire, see e.g.^15^, which is produced by the multiphoton absorption, inverse bremsstrahlung effect and induced avalanche ionization.

**Figure 3:**
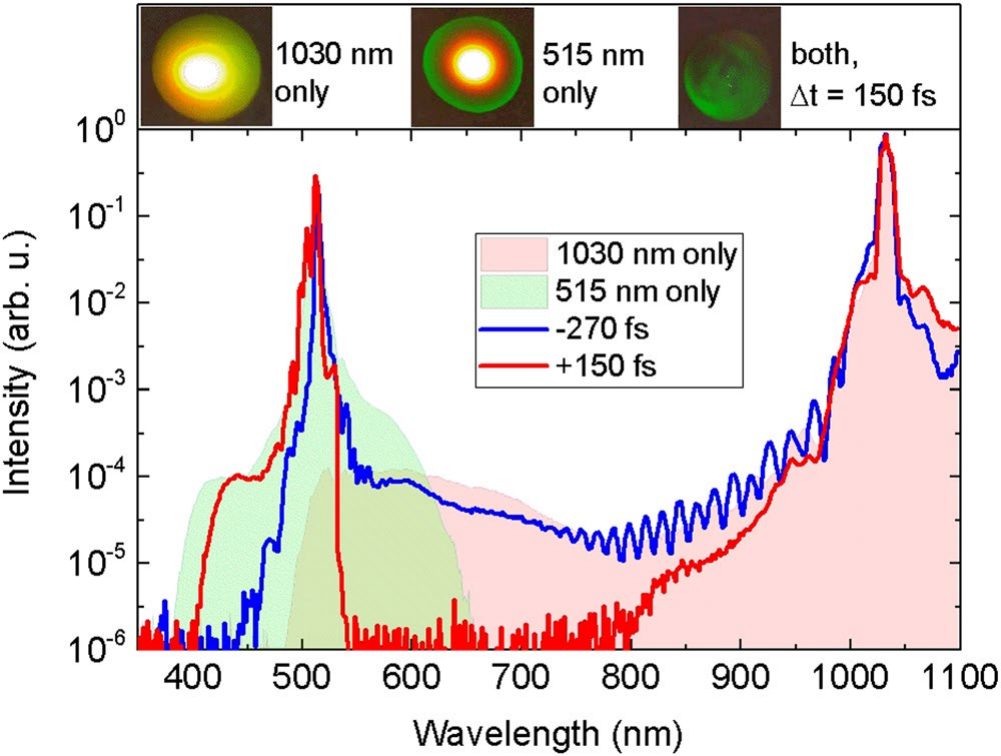
Comparison of SC spectra recorded at delays of −270 fs (blue curve) and +150 fs (red curve). Individual SC spectra produced by fundamental and second harmonic pulses are shaded in red and green, respectively. Insets on the top show the far-field images of SC beam.

When the pulses start to physically overlap in time, the resulting SC spectrum undergoes extremely large transformations, as illustrated in Fig. 3, which presents the most striking examples of modified SC spectra recorded at selected negative and positive delays. More specifically, at a negative delay of −270 fs (i.e. the second harmonic pulse arrives first), suppression of the blue-shifted shoulder of the SC spectrum and occurrence of a distinct modulation of the 700–1000 nm range was observed. The most dramatic change of the SC spectrum occurred at a positive delay of +150 fs (here the fundamental pulse arrives first), where a complete suppression of the spectral components in the region bridging the two pump wavelengths, was recorded. Visually, this suppression was confirmed by an almost complete extinguishing of the SC beam projected on the screen after the 540 nm long-pass filter, as shown in the inset on the right. For a comparison, when any of the pump beams was blocked, stable and bright SC generated by the other beam was observed, as illustrated in the insets on the left and center.

**Figure 4:**
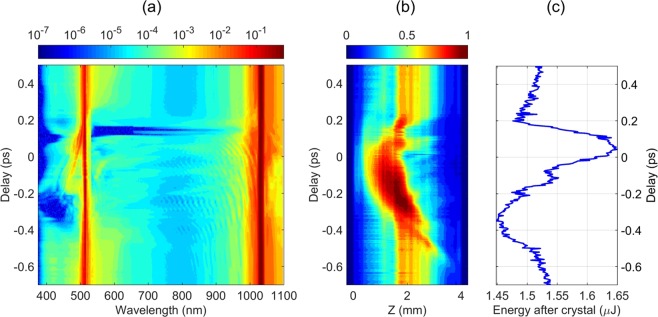
(**a**) SC spectrum, (**b**) integrated intensity of the luminescence track, (**c**) total energy after the crystal as functions of time delay between the fundamental and second harmonic pulses, The input energies of fundamental and second harmonic are 2 *μ*J and 0.4 *μ*J, respectively.

Figure 4(a) provides a bird’s-eye view of the spectral evolution versus the time delay between the incident pulses, which reveals a more complete picture of changes in the resulting SC spectrum. At relatively long delays of <−600 fs and >+200 fs, where the pump pulses do not physically overlap in the crystal, the resulting SC spectra still closely resemble the sum of the SC spectra generated by the pulses independently. The apparent changes in the resulting SC spectrum start to show up for the delay times shorter than −400 fs. In the delay interval from −350 to −200 fs, the most blue-shifted part (350–480 nm) of SC spectrum that is produced by the second harmonic pulse is suppressed completely, whereas distinct spectral modulation appears in the spectral range of 700–1000 nm, where SC originates from the fundamental pulse. As the delay is decreased below −200 fs, the spectral intensity on the short-wavelength side of the SC spectrum recovers, also showing the occurrence of intensified spectral peaks located on both sides of the second harmonic wavelength. The spectral modulation in the 700–1000 nm range persists for a wide range of negative delays and eventually disappears shortly after passing the zero delay. Notice also that there occurs an apparent suppression of the spectral components with wavelengths greater than 1050 nm, although this effect is less pronounced due to reduced sensitivity of the spectrometer. At zero delay, the shape of the resulting SC spectrum is only slightly altered, as compared to the SC spectra recorded at long delays. At positive delays, the spectral changes become a lot more dramatic: the blue shoulder in the 350–480 nm range becomes suppressed starting from +70 fs and recovers at +130 fs, while the visible and near-IR part of the SC spectrum becomes suppressed from +120 fs and recovers only at +200 fs. In a narrow delay region of around +120 fs, the SC spectrum in the UV, visible and near-IR becomes completely extinguished. At +200 fs, the spectral shape of the resulting SC becomes similar to the long-delay spectrum again, although the full recovery of the spectral shape takes until roughly +400 fs. Note that the spectral modulations observed in the near-IR spectral range at negative delays are not observed at positive delays, indicating again, that the time dependence of two-color SC is not symmetric with respect to the time ordering of the pulses.

**Figure 5:**
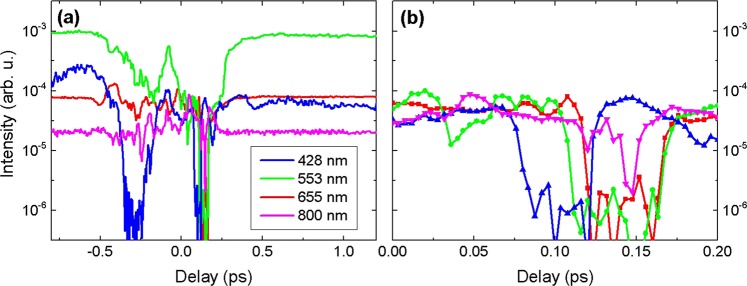
(**a**) Time dependence of SC intensity at 428 nm (blue), 553 nm (green), 655 nm (red) and 800 nm (magenta). (**b**) The same dependence depicted in a reduced temporal window, in a delay range from 0 to +200 fs.

Figure 5(a) illustrates the spectral changes by presenting the spectral intensities versus time delay for the chosen SC spectral components, which are produced by filamentation of second harmonic (428 nm), fundamental (655 and 800 nm) and both pump beams (553 nm). Figure 5(b) highlights very fast time scale of the spectral changes by presenting variations of selected spectral components within a reduced temporal window. A sharp decrease of spectral intensities at 553 and 655 nm is observed in the delay interval from +115 to +170 fs. The transition time from “full SC” to “no SC” is surprisingly short, considering the fact that the duration of pump pulses is 220 fs. The spectral intensities drop by 2.5 orders of magnitude within a change of the delay by 20 fs, and their recovery is equally fast. The spectral intensity at 428 nm also exhibits a similar drop and recovery at somewhat earlier delays (+ 75 fs to +130 fs). The complete suppression of SC is observed at +120 fs delay, where spectral intensities at 428, 533 and 655 nm are diminished to the level of the noise floor of the spectrometer. A slightly different behavior of the spectral intensity at 800 nm, is recorded, which is never suppressed completely, but has two dips at +120 to +145 fs.

In order to get a deeper insight to the observed spectral dynamics, we simultaneously recorded the filament-induced luminescence traces and energy transmittance. Figure 4(b) shows the luminescence intensity integrated across the vertical transverse coordinate, as measured using fine delay step throughout the time window of interest. First of all, notice, that even at long delays, co-filamentation of two beams produces more extended luminescence tracks as compared to those produced by a single beam shown in Fig. 2(b). Interestingly, the initial elongated nonlinear focus produced by two-color filamentation undergoes a sudden jump toward the exit surface of the crystal at ca. −550 fs delay, where the changes in the SC spectrum are still barely detectable. Thereafter the nonlinear focus gradually crawls back towards the entry surface. An apparent increase of the luminescence intensity in the delay range from −350 to −200 fs indicates that here the maximum amount of free electrons is produced. This is exactly the delay interval where the suppression of the most blue-shifted portion of SC spectrum is observed, in line with the findings of ref.^40^. that demonstrate how high density plasma at the nonlinear focus terminates SC generation. By decreasing the delay further on, the peak position of the luminescence trace continues its shift toward the entry face of the crystal, however, its intensity starts gradually dropping. At +50 fs delay the motion of the peak position of luminescence trace reverses its direction, and starts moving back towards the exit surface. In the delay interval from +120 to +160 fs, the intensity of the luminescence track suddenly drops, and its length shrinks significantly. This falls into the delay range, where the visible and near-IR part of the SC is completely suppressed. At longer delays (>+250 fs), the luminescence track reverts to its original intensity and profile along with the recovery of the SC spectrum.

Figure 4(c) presents the energy measured after the crystal, which also exhibits a clear dependence on the relative timing of the two pump pulses. At large delays, we measured a decrease of the total incident energy from 2.0 *μ*J to 1.54 *μ*J. The energy losses are contributed by multiphoton and plasma absorption, experienced by each pump pulse independently, and by Fresnel reflections from the input and output faces of an uncoated sapphire sample. As the pulses start to interact, the transmitted energy decreases below the value of 1.54 *μ*J measured at long negative delays, reaches a minimum of 1.46 *μ*J at −300 fs delay, then starts increasing, and reaches a maximum of 1.65 *μ*J at +50 fs. Subsequently, the energy drops again to reach another local minimum of 1.48 *μ*J at +200 fs, and slowly settles to a new equilibrium value of 1.52 *μ*J. The total swing of the output energy constitutes ~12% of the initial energy value. Two relevant aspects are worth noting in the energy dependence: first, the initial and final energies are slightly different, showing once again that the medium retains some “memory” of the pulse for at least a couple of picoseconds; second, the extremes of the transmitted energy do not exactly coincide with relevant changes of the SC spectra and luminescence traces. This is clear from the inspection of Fig. 4, indicating the delay positions where specific SC spectra are observed, apparently fall on the slopes of the energy variation curve.

**Figure 6:**
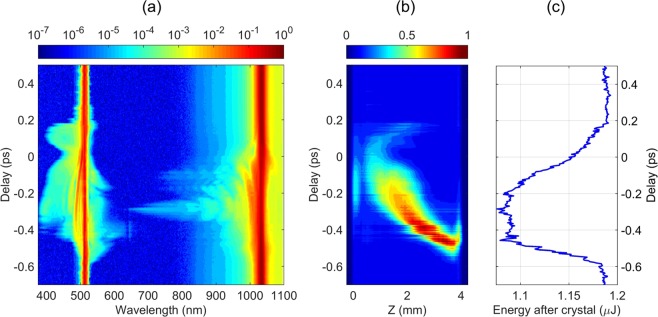
The same as in Fig. 4, just the energies of fundamental (1.32 *μ*J) and second harmonic (0.24 *μ*J) pumps are set below individual filamentation and SC generation thresholds.

**Figure 7:**
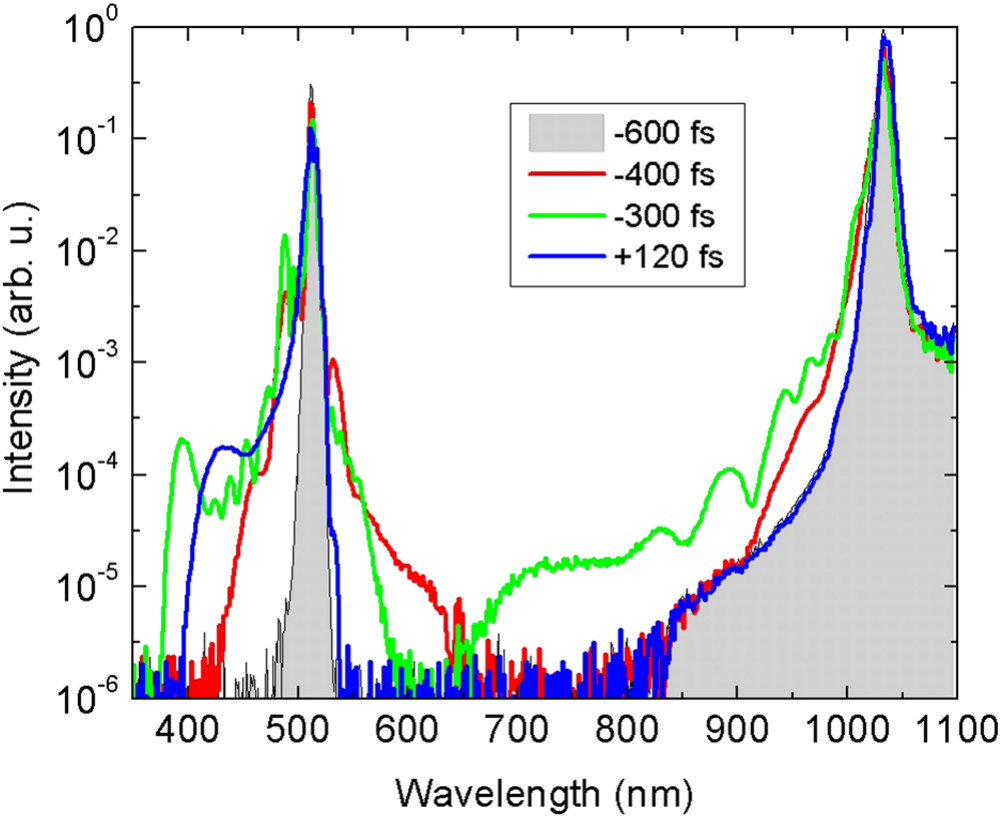
Comparison of the output spectra recorded at particular delays when the individual input energies of fundamental and second harmonic pumps were set below filamentation threshold (1.32 *μ*J at 1030 nm and 0.24 *μ*J at 515 nm).

Interestingly, the interaction between the input pulses leading to co-filamentation and SC generation was observed when the energies of both pump pulses were decreased below their individual filamentation and SC generation thresholds. Figure 6(a) shows spectral evolution as a function of delay between the fundamental and second harmonic pulses with the input energies of 1.32 *μ*J and 0.24 *μ*J, respectively. Considerable spectral broadening around the second harmonic wavelength starts to be observable at relatively large delays, i.e. from −550 fs, at roughly −350 fs delay, the SC lights up in the entire spectral range from 380 to 1030 nm. At zero delay, the SC disappears again, and at ca. +170 fs only the blue-shifted portion of the spectrum appears for the second time and returns back to “no SC” situation at ca. +200 fs. Figure 7 presents the illustrative lineouts of the output spectra in the absence (−600 fs) and in the presence (−400, −300 and +120 fs) of co-filamentation, as retrieved from the spectral map shown in Fig. 6(a).

The variation of luminescence intensity versus delay is presented in Fig. 6(b) and attests the relationship between spectral broadening and onset of co-filamentation; the absence of luminescence for large negative and positive delays indicates no filamentation. The characteristic features of luminescence intensity (the shift of peak position and variation of intensity) are qualitatively similar to those presented in Fig. 4(b). The measured energy transmittance is presented in Fig. 6(c). A drop in the transmitted energy, which is associated with the onset of nonlinear losses, clearly marks the time window (from −550 to +200 fs) of co-filamentation that fairly coincides with the delay range of physical overlap of pump pulses inside the crystal. However, notice that in the present case, the measured energies at large negative and positive delays are equal, so no “memory” in the absence of filamentation could be detected.

## Discussion

To summarize the above observations, we demonstrated that co-filamentation of two color femtosecond laser pulses produces a non-trivial nonlinear interaction in the medium. Experimentally, this manifests itself as a complex behavior of the resulting spectrum, position and intensity of the filament-induced luminescence track, and energy transmittance with the following interesting features that need to be highlighted:

- When both pulses carry the energies above filamentation and SC generation threshold, their interaction is mainly destructive with respect to SC generation. The opposite is true for the case when both pulses carry the energies below filamentation and SC generation threshold.
- SC generation goes from “fully on”to “fully off” and back within the change of the delay just by 20 fs, despite the fact that both pump pulses are relatively long (220 fs).
- There is an obvious correlation between the SC spectrum, position of the nonlinear focus, intensity distribution along the filament-induced luminescence traces and transmitted energy versus the time delay, and asymmetry with respect to time ordering of the pulses.

The last observation could be intuitively explained by the fact that fundamental and second harmonic pulses travel with different group velocities, *v*_FH_ = 0.5636*c* and *v*_SH_ = 0.5512*c*, as estimated taking the group refractive indices of 1.7744 and 1.8143 at 1030 nm and 515 nm, respectively. This yields the group velocity mismatch length of 133 fs/mm, meaning that the incident 220 fs fundamental and second harmonic pulses separate by the time interval equal to their duration after 1.65 mm of propagation in sapphire crystal. As the second harmonic pulse travels slower than fundamental, when it enters the medium first (the case of negative delays), the fundamental pulse is catching up with it, and both pulses temporally overlap inside the nonlinear medium. On the contrary, when the fundamental pulse enters first (the case of positive delays), it always escapes from the second harmonic, in the simplest approximation, leaving only the affected medium for the second harmonic pulse to interact with.

The relevant features of the spectral dynamics could be qualitatively interpreted in terms of the group velocity difference between the fundamental and second harmonic pulses and pulse splitting-based SC generation scenario in a normally dispersive medium^41^. This scenario implies that SC is produced by the pulse splitting, which is the space-time effect and occurs at the nonlinear focus. The split sub-pulses subsequently undergo self-steepening of the pulse fronts due to the interplay between the nonlinear effects and chromatic dispersion, inducing sharp intensity gradients (optical shocks) in the temporal profiles of sub-pulses, which give rise to an explosive broadening of the spectrum, i.e. SC generation, see illustrative numerical examples provided in ref.^36^. More precisely, the red-shifted frequencies are generated by self-steepening of the ascending front of the leading sub-pulse, whereas the blue-shifted frequencies are generated by self-steepening of the descending front of the trailing sub-pulse. As a result, the leading and trailing sub-pulses have different carrier frequencies and propagate with different group velocities^42^.

The above SC generation scenario applies to fundamental and second harmonic pulses, since both lie in the region of normal GVD of sapphire. Since the two pump pulses then co-propagate in a nonlinear medium, they act on each other and experience self-action, the observed delay dependence could be understood in the frame of a time-varying distortion of the refractive properties of the nonlinear medium via Kerr effect and generation of free electron plasma. If the presence of one pulse creates a distortion of the refractive properties of the crystal, self-focusing of the other pulse may be strongly affected by it, altering the overall filamentation dynamics. While the suppression of SC generation is most obvious in the data, note that the signatures of amplification of particular spectral portions around the second harmonic wavelength are also discernible, e.g. in the delay range between −100 fs and +100 fs. In addition, the intensity modulation in the spectral range bridging the two pump wavelengths is observed within a broad range of negative delays, appearing as distinct wavelets in Fig. 4(a). This modulation closely resembles the interference pattern that occurs in the SC spectrum produced by a single pulse due to filament refocusing at higher energies (Fig. 2), suggesting that presence of the second harmonic pulse induces refocusing of a filament at fundamental wavelength and subsequent pulse splitting. In principle, multiple refocusing events should be visible in the luminescence traces in Fig. 4(b), however, in the case of co-filamentation, the integrated intensities are too blurred to allow definite conclusions.

An important inference can be made from the change of the intensity and the shape of luminescence track observed at time delays where large portions of the SC spectrum are “turned off” (Fig. 4). In particular, in the delay interval from +120 fs to +160, where the blue wing of SC generated by the fundamental pulse and the red wing of SC generated by the second harmonic pulse are suppressed, the luminescence track becomes considerably fainter and distinctly shorter, as compared to the neighboring delay points where changes in the SC spectrum are small. It thus seems that the two pulses still induce a filament, but their interaction disturbs pulse front steepening rather than halts the entire pulse splitting event. This is particularly evident from the extinction of certain portions of the SC spectrum associated with a particular sub-pulse (e.g. suppression of the spectral components in the 380–500 nm range at around −250 fs and +120 fs delays and at wavelengths greater than 1050 nm in the delay interval from −350 fs to 0 fs). Ability to affect subpulses separately indicates that the presence of two input pulses can still alter the pulse dynamics after the splitting event, i.e. it can affect pulse front steepening stage rather than preventing the initial collapse of the beam into nonlinear focus. The extremely steep fronts of sub-pulses could, in principle, be invoked to explain very short times in which SC is suppressed and appears again as the delay between pulses is varied. This idea could, in principle, be tested experimentally by time-domain measurements such as frequency-resolved optical gating. However, practical implementation of such experiment is extremely challenging for two reasons: first, both pump wavelengths fall in the normal GVD region of the material, implying that both pulses undergo splitting. Therefore, a total of four broadened pulses emerge from the nonlinear medium. This complicated temporal structure, in combination with extremely broad spectra would make the measurement and reconstruction of time-domain data a non-trivial task. Secondly, pulse front steepening takes place immediately after the pulse splitting at the nonlinear focus and the overall process is confined within a short propagation distance. Thereafter material dispersion acts on the ultrabroad spectrum of the pulse, and dispersive broadening destroys its original steep fronts. Since in our experiments the nonlinear focus is located well inside the nonlinear material (as the positions of the luminescence tracks suggest), the pulse(s) at the output of the crystal will be dispersively broadened, and the steep fronts will no longer be detectable.

The transmitted energy dependence on the delay between pump pulses suggests that pulse timing significantly affects the amount of absorbed energy. The dominant mechanism changing the transmittance of the medium is the nonlinear absorption in the vicinity of the nonlinear focus and absorption of free electron plasma by inverse bremsstrahlung effect. However, it is still not clear why the most dramatic changes in the output spectra occur at the slopes rather than at the extremes of the energy transmittance curve.

The case where both beams have insufficient energy for SC generation is also insightful. Correlation between spectral broadening, occurrence of luminescence tracks and drop in energy transmittance (Fig. 6) clearly show that at the appropriate delays, the weaker pulses “collaborate” helping each other to self-focus and generate SC.

## Conclusions

To summarize, we demonstrated that co-filamentation of two color femtosecond laser pulses in sapphire yields dramatic changes in the SC spectrum, position of the nonlinear focus, intensity distribution along the filament-induced luminescence traces and transmitted energy when varying the time delay between the incident pump pulses. In particular, we show that at certain delays some portions of the SC spectrum are amplified, whilst at the other delays, large portions of the SC spectrum are completely extinguished, although each of the pump pulses launched separately produces SC with a smooth spectrum. The transition between SC generation and its extinction at a particular spectral range occurs within a very short (20 fs) change of the delay time, despite the fact that the pump pulses are 220 fs long. A non-trivial nonlinear interaction is explained in terms of the group velocity difference between the two color pump pulses and pulse splitting-based SC generation scenario in a normally dispersive nonlinear medium. We propose that the observed spectral features can be interpreted by a mechanism, where co-propagating two pump pulses perturb the nonlinear refractive properties of the medium via Kerr effect and generation of free electron plasma thereby affecting pulse splitting and pulse front steepening, which are the key players in the process of supercontinuum generation. Almost identical spectral dynamics versus the time delay, i.e. amplification and extinction of certain portions of SC spectrum, were recorded in a YAG crystal (see Supplementary Fig. S2), proving that the uncovered interaction mechanism is rather universal.

## Supplementary information

SUPPLEMENTARY INFORMATION for Supercontinuum generation by co‐filamentation of two color femtosecond laser pulses
