# Supplementary material for: Supercontinuum generation by co-filamentation of two color femtosecond laser pulses
Source: Sci Rep. 2019 Jun 21;9:9011. doi: 10.1038/s41598-019-45357-y (PMC6588607; doi:10.1038/s41598-019-45357-y)
Supplement: Supplementary file 1 — SUPPLEMENTARY INFORMATION for Supercontinuum generation by co‐filamentation of two color femtosecond laser pulses [file 41598_2019_45357_MOESM1_ESM.pdf]

SUPPLEMENTARY INFORMATION

Supercontinuum generation by co-filamentation of two color femtosecond laser pulses

M. Vengris, N. Garejev, G. Tamošauskas, A. Čepėnas, L. Rimkus, A. Varanavičius, V. Jukna, and A. Dubietis

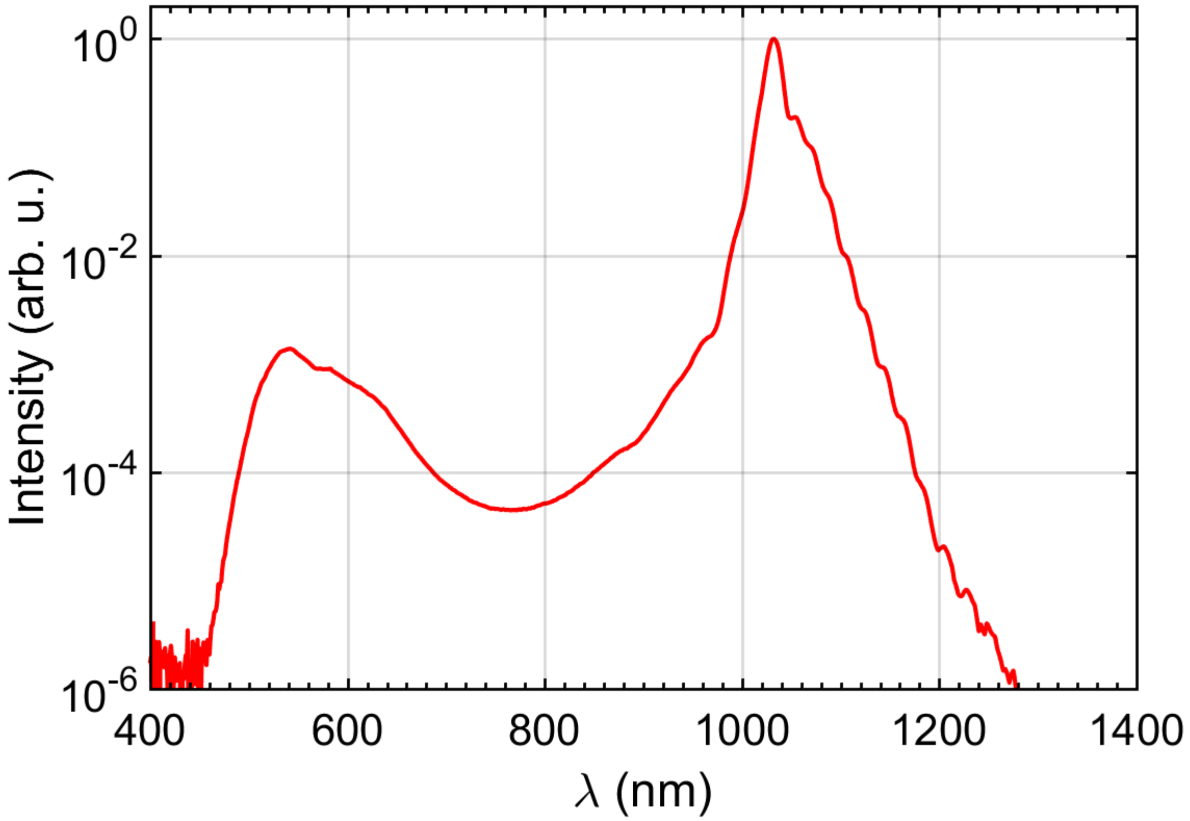

Supplementary Fig. S1. Full extent of SC spectrum generated by filamentation of 220 fs, 1030 nm laser pulse with an energy of 2 μJ in a sapphire crystal of 4 mm thickness.

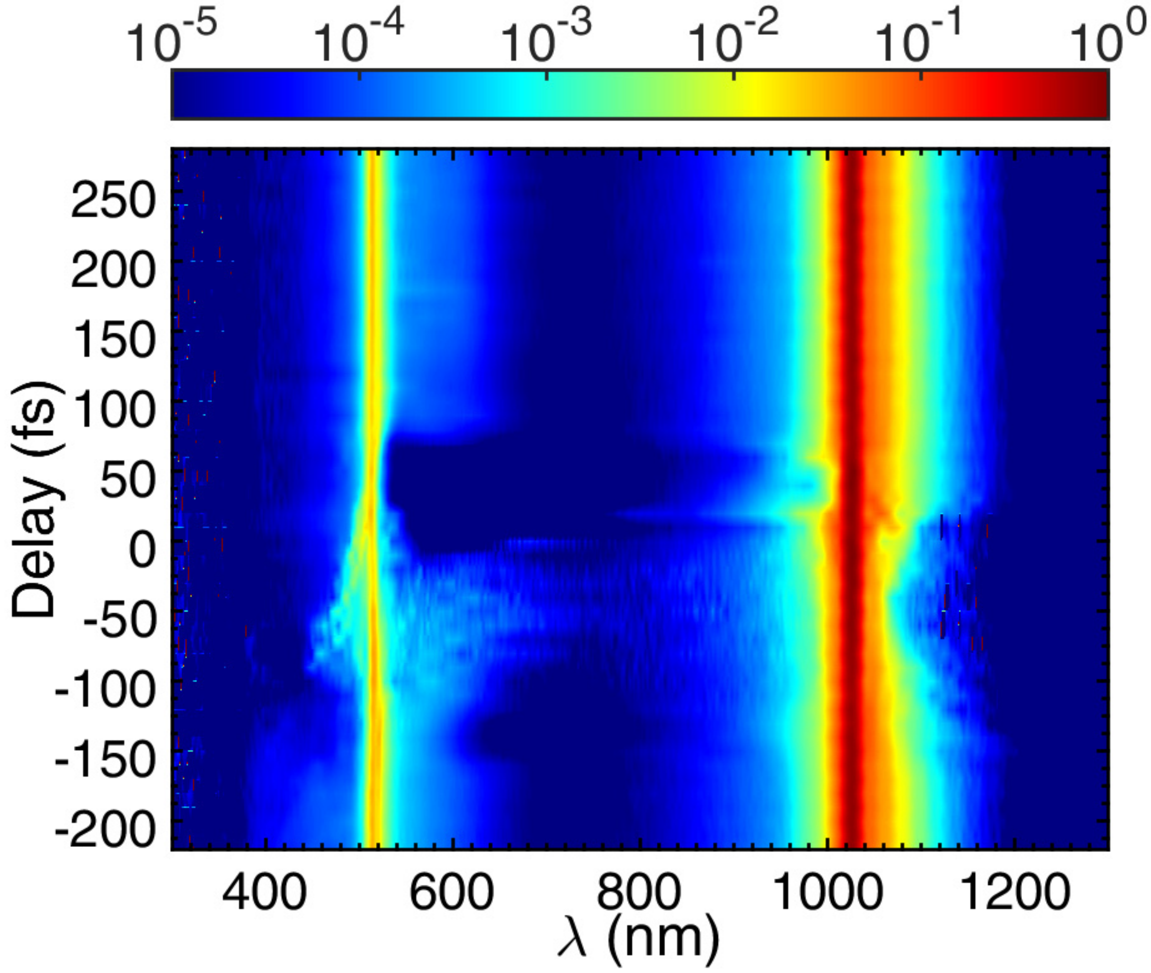

Supplementary Fig. S2. SC spectrum as function of time delay between the fundamental and second harmonic pulses in a YAG crystal of 4 mm thickness. The input pulse energies of fundamental and second harmonic pulses are 1.24 μJ and 0.2 μJ, respectively. Note that the delay range where relevant spectral changes occur is narrower as compared to that in sapphire due to larger group velocity mismatch between the pump pulses in YAG.
